# The impact of social and physical distancing measures on COVID-19 activity in England: findings from a multi-tiered surveillance system

**DOI:** 10.2807/1560-7917.ES.2021.26.11.2001062

**Published:** 2021-03-18

**Authors:** Jamie Lopez Bernal, Mary A Sinnathamby, Suzanne Elgohari, Hongxin Zhao, Chinelo Obi, Laura Coughlan, Vasileios Lampos, Ruth Simmons, Elise Tessier, Helen Campbell, Suzanna McDonald, Joanna Ellis, Helen Hughes, Gillian Smith, Mark Joy, Manasa Tripathy, Rachel Byford, Filipa Ferreira, Simon de Lusignan, Maria Zambon, Gavin Dabrera, Kevin Brown, Vanessa Saliba, Nick Andrews, Gayatri Amirthalingam, Sema Mandal, Michael Edelstein, Alex J Elliot, Mary Ramsay

**Affiliations:** 1 Public Health England COVID-19 Surveillance Cell, London, United Kingdom; 2 Department of Computer Science, University College London, London, United Kingdom; 3 Public Health England COVID-19 Virology Cell, London, United Kingdom; 4 Nuffield Department of Primary Care Health Sciences, University of Oxford, Oxford, United Kingdom; 5 Royal College of General Practitioners (RCGP) Research and Surveillance Centre (RSC), London, United Kingdom; 6 Public Health England COVID-19 Epidemiology Cell, London, United Kingdom

**Keywords:** social distancing, non-pharmaceutical interventions, lockdown, surveillance, COVID-19, coronavirus

## Abstract

**Background:**

A multi-tiered surveillance system based on influenza surveillance was adopted in the United Kingdom in the early stages of the coronavirus disease (COVID-19) epidemic to monitor different stages of the disease. Mandatory social and physical distancing measures (SPDM) were introduced on 23 March 2020 to attempt to limit transmission.

**Aim:**

To describe the impact of SPDM on COVID-19 activity as detected through the different surveillance systems.

**Methods:**

Data from national population surveys, web-based indicators, syndromic surveillance, sentinel swabbing, respiratory outbreaks, secondary care admissions and mortality indicators from the start of the epidemic to week 18 2020 were used to identify the timing of peaks in surveillance indicators relative to the introduction of SPDM. This timing was compared with median time from symptom onset to different stages of illness and levels of care or interactions with healthcare services.

**Results:**

The impact of SPDM was detected within 1 week through population surveys, web search indicators and sentinel swabbing reported by onset date. There were detectable impacts on syndromic surveillance indicators for difficulty breathing, influenza-like illness and COVID-19 coding at 2, 7 and 12 days respectively, hospitalisations and critical care admissions (both 12 days), laboratory positivity (14 days), deaths (17 days) and nursing home outbreaks (4 weeks).

**Conclusion:**

The impact of SPDM on COVID-19 activity was detectable within 1 week through community surveillance indicators, highlighting their importance in early detection of changes in activity. Community swabbing surveillance may be increasingly important as a specific indicator, should circulation of seasonal respiratory viruses increase.

## Introduction

The United Kingdom (UK) was one of the earliest countries in Europe to experience importations of coronavirus disease (COVID-19), with the first cases detected at the end of January 2020 [1,2]. Following a steady increase in case numbers during March 2020, the government introduced a range of measures to limit transmission in the community. On 12 March 2020 (week 11), individuals with a continuous cough or fever were advised to self-isolate for 7 days, school trips abroad were cancelled and at risk groups were advised to avoid cruises [3]. On 16 March (week 12), the government advised against non-essential travel and contact with others, and advised on working from home where possible [4]. Mandatory social and physical distancing measures (SPDM) were then introduced from 23 March 2020 (week 13) and included: closing schools except for socially vulnerable children and children of critical workers, requiring people to stay at home except for very limited purposes, closing certain businesses and venues, and stopping all gatherings of more than two people in public [5].

**Figure 1:**
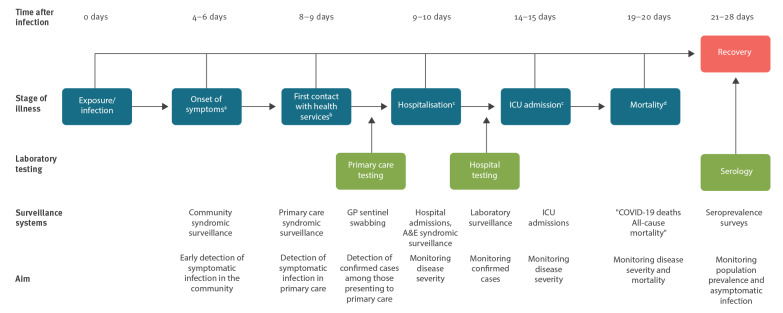
Average time from infection with SARS-COV-2 to different stages of illness and levels of care that individuals may experience

Reducing the level of contact between individuals is intended to reduce the effective reproduction number (Re) to below one so that one individual infects less than one other person and the epidemic declines. It was anticipated that social and physical distancing interventions would first reduce the number of exposures to confirmed cases, which would in turn reduce the number of new infections, presentations to healthcare services and fatalities. The expected time from the introduction of social and physical distancing measures to a detectable impact on surveillance indicators is estimated based on the incubation period and typical time from symptom onset to the different stages of illness and levels of care that individuals may experience (Figure 1).

A&E: accident and emergency department; COVID-19: coronavirus disease; GP: general practice; ICU: intensive care unit; SARS-CoV-2: severe acute respiratory syndrome coronavirus 2.

^a^ Incubation period as reported by the World Health Organization [7].

^b^ Time from symptom onset to first contact with health services is based on the UK Flusurvey COVID-19 collect [11].

^c^ Time from symptom onset to hospitalisation and ICU admission is based on the COVID-19 hospitalisation in England surveillance system (CHESS).

^d^ Time from symptom onset to death is based on the UK first few hundred (FF100) study [13].

The Public Health England (PHE) COVID-19 surveillance systems are predominantly built on existing surveillance systems for influenza and aim to monitor the burden of COVID-19 at different points in the course of illness and at different levels of care. These include seroprevalence surveys to estimate rates of infection; population surveys, web searches and syndromic surveillance to detect symptomatic infection in the community; sentinel swabbing to detect confirmed cases among those presenting to primary care; hospital surveillance of confirmed cases at different levels of care; surveillance of deaths among confirmed COVID-19 cases and excess all-cause mortality to monitor severity outcomes; and serosurveillance and mass testing to detect asymptomatic infection (A graphical overview of the surveillance systems is presented in Supplementary Figure 1).

Survey evidence suggests that SPDM in the UK have substantially reduced contact levels [6]. Here, we report the impact of SPDM on COVID-19 activity in England, how this impact manifested in the various surveillance systems and how the surveillance systems may be applied to detect increases in COVID-19 activity as SPDM are eased.

## Methods

Data from a range of PHE surveillance systems are used to assess the impact of the SPDM by comparing the timing of peaks in COVID-19 activity to the expected lags from the introduction of the measures based on the time from infection to different stages of illness and levels of care as outlined in Figure 1. These calculations are based on an estimated COVID-19 incubation period of 4–6 days (range 1–14 days), and time from symptom onset to presentation at different levels of care [7,8]. The surveillance systems used are described below. Further details are available elsewhere [9,10]. Data starting from week 1 2020 (where available) to week 18 2020 are included.

### Community and primary care surveillance

Indicators of infection in the community are based primarily on surveillance of respiratory symptoms through population surveys of self-reported symptoms, frequency of web searches on COVID-19 symptoms and reporting of respiratory syndromes during first contact with health services, including primary care services and contacts with the National Health Service (NHS) telephone and Internet medical advice line (NHS 111).

### Population surveys

Flusurvey, part of the European wide Influenzanet initiative, is a weekly web-based symptom survey with approximately 4,500 participants in total, which was originally set up during the 2009 influenza H1N1 pandemic [11]. Participants answer a series of questions about respiratory symptoms, exposure risk and healthcare-seeking behaviour. For the purposes of this study, we report rates of fever or cough as an indicator of COVID-19 activity in the community.

### Web search queries

A web-based syndromic surveillance system for COVID-19 was developed in 2020 by Lampos et al. [12] using daily search query frequency statistics obtained from the Google Health Trends API. This unsupervised model focuses on search queries about COVID-19 symptoms as identified by the first few hundred (FF100) questionnaire [13], as well as generic queries about coronavirus (e.g. COVID-19). The search query frequency time series is standardised and weighted based on symptom frequency as reported in the UK FF100 study [13]. In addition, queries about the symptom of anosmia are also incorporated. This time series is also weighted for media debiasing to minimise bias in the search query, which is due to public interest rather than disease itself. Frequency of searches for symptoms was compared with a baseline calculated from historical daily data from October 2011 to September 2019. Confidence intervals for the baseline data were calculated in order to identify a departure from the expected number of searches during the current time period.

### Syndromic surveillance

PHE’s real-time syndromic surveillance team coordinate daily collection and analysis of respiratory syndromic indicators at different levels of care including NHS 111 calls, general practice (GP) in hours and out of hours contacts, ambulance dispatch calls and accident and emergency department (A&E) visits [14]. Clinical indicators are also collected by the Royal College of General Practitioners Research and Surveillance Centre (RCGP RSC), a network of general practices that contribute electronic health record data on primary care consultations, a proportion of which also contribute respiratory swabs to the national reference laboratory (discussed below) [15]. Of particular importance to the network is reliable recording of influenza-like illness (ILI). New COVID-19 specific syndromic indicators have recently been developed in both of these syndromic collections to monitor activity through NHS 111, GP and A&E systems [14]. For example, a new surveillance indicator was developed to capture GP consultations using new codes for suspected, tested, exposed and confirmed COVID-19 cases. The COVID-19 epidemic has also led to changes in guidance on where the public should seek healthcare in England, as well as changes to coding of respiratory syndromes through electronic health record systems, both of which have had artefactual impacts on syndromic surveillance indicators. For example, changes in national coding and clinical pathways within the NHS 111 telephone system (introduced to support the triage of potential COVID-19 patients) resulted in callers with COVID-19 symptoms no longer being reported through existing NHS 111 respiratory syndromic surveillance call pathways. For each syndromic surveillance indicator, 7 day moving averages are calculated based on the preceding 7 days.

### Primary care sentinel swabbing

Sentinel nasal swabbing of patients contacting primary care with ILI (acute respiratory illness with fever and cough) or acute lower respiratory tract infection (LRTI) symptoms (GP-diagnosed) was initially conducted through 100 practices in the RCGP RSC network, though this network was subsequently expanded to 300 to provide improved coverage. To be included, swabs should be taken within 7 days of symptom onset. This system has been expanded and modified to adapt to recommendations that symptomatic individuals should not visit their GP, but instead use practices operating telephone and/or video consultations. Here, participants undertake a self-swab, sent to them via post, of both nostrils, and return the swab to the national reference laboratory for PCR testing and further characterisation. Onset date is collected through a patient-completed sample request form, and positivity is reported by symptom onset date. RCGP RSC practices receive feedback about data quality, including via a dashboard [16].

### Respiratory outbreaks

Respiratory outbreaks are managed by local PHE Health Protection Teams. These are detected either by identifying clusters of cases through laboratory surveillance systems or by direct reporting from the outbreak setting to the Health Protection Team. Data on all acute respiratory illness incidents by setting (e.g. nursing homes, schools) are collected through the public health management system (HPZone) used by local Health Protection Teams. This includes both suspected and confirmed COVID-19 outbreaks.

### Secondary care

Acute NHS hospital trusts were asked to report aggregate data on daily new hospitalisations and critical care (ICU/HDU) admissions for COVID-19 to the COVID-19 hospitalisations in England surveillance system (CHESS).

During the period of SPDM, most laboratory testing for COVID-19 occurred in hospitals. Therefore, rates of laboratory confirmed cases also provide an indication of activity in hospitals. Testing in PHE and NHS laboratories is reported to PHE through the second generation surveillance system (SGSS) [17]. This system has been adapted to capture negative as well as positive results. Number of positive cases or rates per 100,000 population are influenced by testing capacity as well as disease activity. Positivity rates (as a proportion of all tests) are less influenced by testing capacity and provide a more reliable measure of disease activity, although they could still be influenced by changes in policy on which groups are eligible for testing.

Median time from symptom onset to first hospital admission and interquartile ranges were estimated based on the individual level reporting.

### Mortality

Mortality surveillance includes data on deaths among laboratory confirmed COVID-19 cases (which will primarily be hospitalised cases) and excess all-cause mortality. Daily excess all-cause mortality is estimated using deaths data from the General Register Office, which are delay corrected (based on past data on delay to registration from death) and compared to a baseline calculated from the previous 5 years. Weekly excess all-cause mortality is estimated using the EuroMomo model [18], and presented as z-scores.

### Ethical statement

The surveillance collections included here are approved as Health Protection under Regulation 3 of The Health Service (Control of Patient Information) Regulations 2002.

## Results

### Infection in the community

#### Population surveys and web search queries

**Figure 2:**
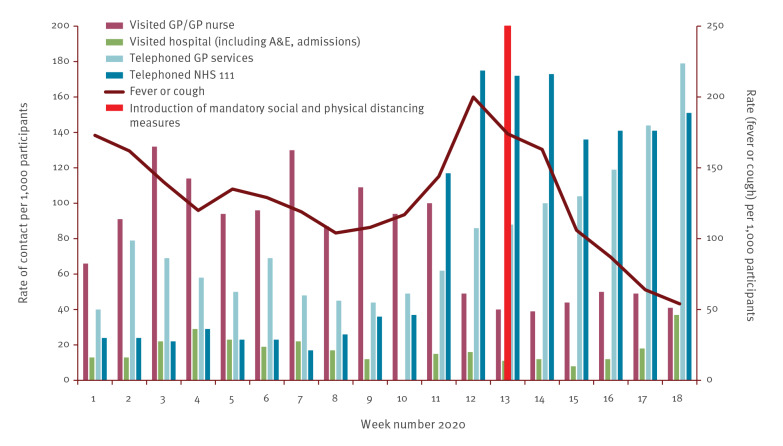
Rate of fever or cough reported by Flusurvey participants and their contact with different healthcare services^a^, United Kingdom, week 1 2020 to week 18 2020

Assuming the SPDM had an immediate impact on exposure, and that a relatively large proportion of reported symptoms were due to COVID-19, we would expect to start seeing an impact on self-reported symptoms within one incubation period of the measures being introduced. This is in line with the reporting of a fever or cough via Flusurvey, which began to decline in week 13 (Figure 2). This was also seen when a more specific definition of ‘Fever and cough’ was used (further details in Supplementary Figure S2). We also saw a reduction in visits to general practices.

A&E: accident and emergency department; GP: general practice; NHS 111: national health service medical advice line.

^a^ Flusurvey captures data if the participant states they have telephoned the NHS 111 service, but not if they state they have used the NHS 111 Internet medical advice line.

**Figure 3:**
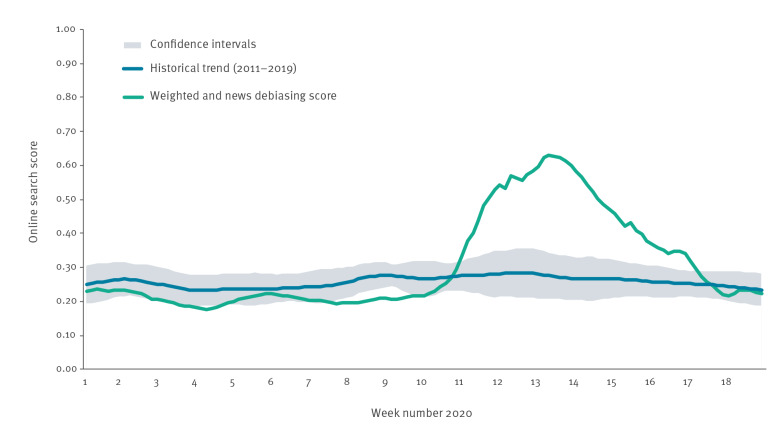
Standardised Google search score for COVID-19 symptoms, with weighted score for media debiasing and historical trend, United Kingdom, week 1 2020 to week 18 2020

Google searches for COVID-19 symptoms began to decline from 28 March, 5 days after the introduction of SPDM, suggesting that SPDM had had a rapid effect on symptomatic infection rates (Figure 3).

COVID-19: coronavirus disease.

#### Syndromic surveillance

Flusurvey responses showed that the earliest contact with health services was among those who called NHS 111 (median 2 days), suggesting that we would expect to see an early impact on NHS 111 calls if SPDM were effective (further details in Supplementary Table 1). Changes to the NHS 111 coding and care pathways resulted in a rapid drop in reported NHS 111 calls through these existing respiratory surveillance indicators (further details in Supplementary Figure S3). These changes in reporting coincided with the introduction of SPDM.

**Figure 4:**
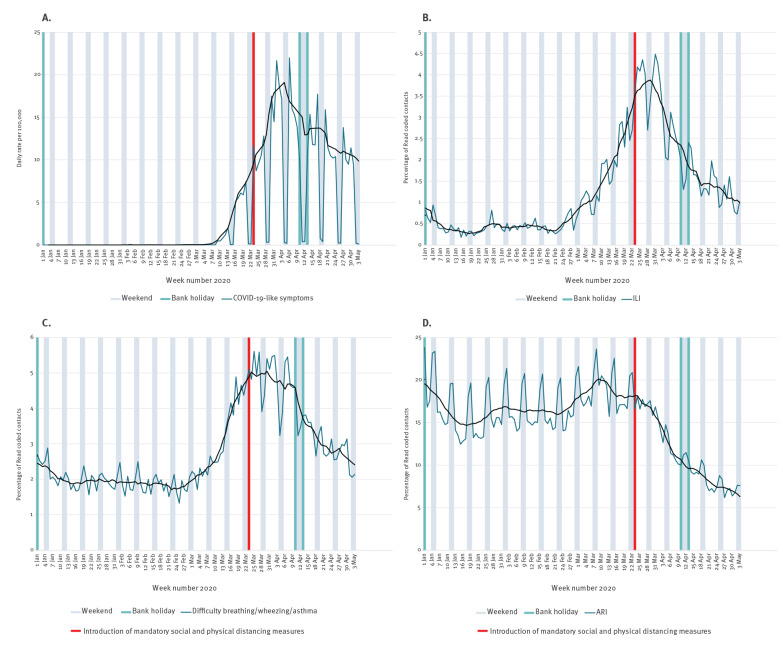
The effect of mandatory social and physical distancing measures on syndromic surveillance indicators (A) COVID-19 GP diagnosis code indicators^a^ (B) daily GP out of hours contacts with a diagnosis code for influenza-like illness, (C) daily GP out of hours contacts with a diagnosis code for difficulty breathing/wheezing/asthma, (D) daily GP out of hours contacts with a diagnosis code for acute respiratory infection, United Kingdom, week 1 2020 to week 18 2020

ARI: acute respiratory infection; COVID-19: coronavirus disease; GP: general practice; ILI: influenza-like illness.

^a^ Includes consultations using new codes for suspected, tested, exposed and confirmed COVID-19.

Current guidelines recommend remote consultations for suspected COVID-19 cases in primary care [19]. The majority of phone calls to GP practices reported through Flusurvey were within the first week of SPDM (median 5 days; further details in Supplementary Table S1). Allowing for the incubation period, we would expect to see an impact on GP contacts from 1 to 2 weeks after the introduction of SPDM. The new COVID-19 consultation indicator began to decline 12 days after the mandatory SPDM were introduced (Figure 4). GP out of hours (OOH) syndromic indicators were relatively unaffected by changes to guidance and clinical coding. OOH syndromic data suggest that rates of contacts for influenza like illness (ILI) and difficulty breathing/wheezing/asthma began to decline from 7 days and 2 days after the introduction of mandatory SPDM, respectively. However, contacts for the less specific acute respiratory infection indicator peaked in week 11, which preceded the introduction of mandatory SPDM, though this peak was at much lower levels compared with the winter peak (Figure 4).

Red vertical lines indicate the introduction of mandatory social and physical distancing measures.

Black line indicates 7 day moving average (adjusted for bank holidays).

The median time to A&E visits among Flusurvey participants was 4 days. We would therefore expect an impact of SPDM on A&E attendances within 1–2 weeks. A&E attendances with acute respiratory infections and COVID-19 related primary diagnosis codes began to decline 13 and 14 days after the introduction of mandatory SPDM, respectively (further details in Supplementary Figure S5).

#### Primary care sentinel swabbing

**Figure 5:**
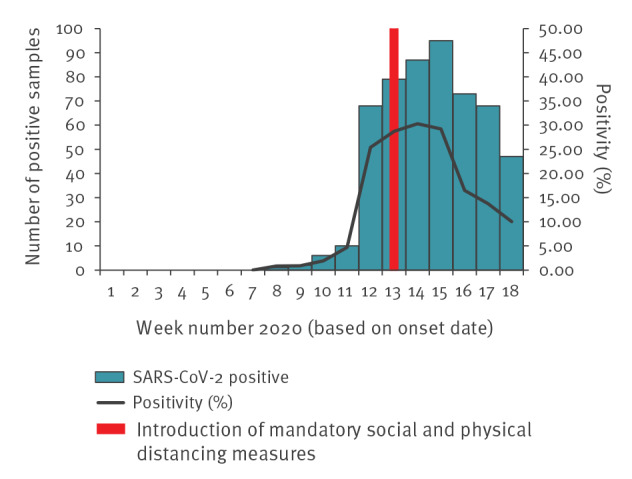
Number of SARS-CoV-2 positive samples and overall positivity rate of weekly GP sentinel swabs, United Kingdom, week 5 2020 to week 20 2020

It is anticipated that any impact on the SARS-CoV-2 positivity of primary care sentinel swabs by symptom onset date would occur within one incubation period of the introduction of SPDM. There was a decline in the rate of increase in positivity from week 12 to week 13 and positivity rates peaked in week 14, which was in accordance with an impact of SPDM social distancing (see Figure 5, with further details in Supplementary Figure S7).

SARS-CoV-2: Severe acute respiratory syndrome coronavirus 2.

### Respiratory outbreaks

**Figure 6:**
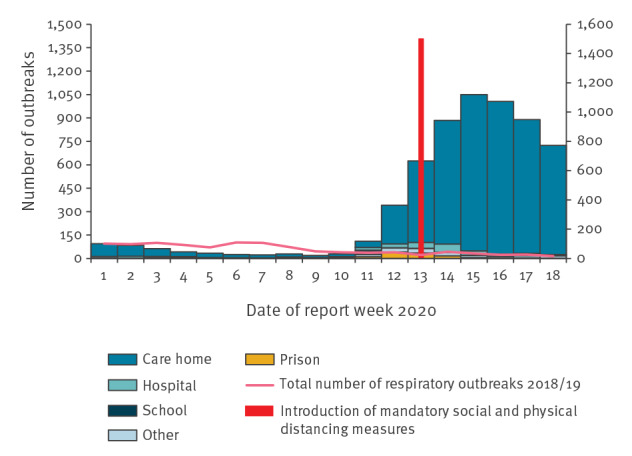
Number of acute respiratory outbreaks by institution, United Kingdom, week 1 2020 to week 19 2020

The number of reported acute respiratory infection outbreaks increased dramatically after week 12, the majority of which were suspected or confirmed COVID-19 outbreaks in nursing homes. Outbreaks began to decline from week 16. However, the number of outbreaks remained high into week 18, suggesting that SPDM may have less of an impact or a more delayed impact on outbreaks in residential settings (Figure 6).

#### Secondary care

**Figure 7:**
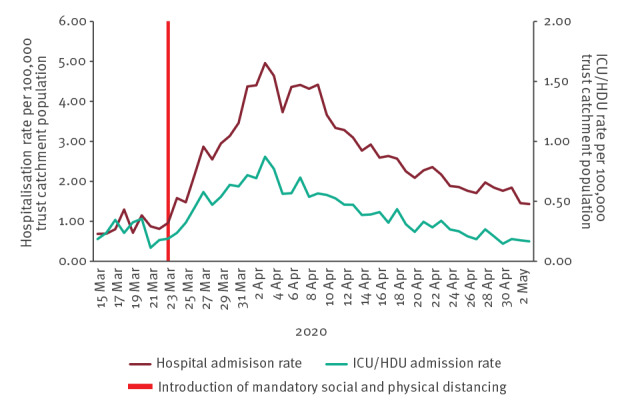
Daily hospital admission rates and critical care^a^ admission rates of patients with confirmed SARS-CoV-2, United Kingdom, week 11 2020 to week 18 2020

HDU: high dependency unit; ICU: intensive care unit; SARS-CoV-2: severe acute respiratory syndrome coronavirus 2.

^a^ HCD/ICU admissions.

In the individual level hospitalisation data, median time from symptom onset to hospital admission was 4 days (IQR: 1–8 days) and median time from symptom onset to ICU admission was 9 days (IQR: 4–12 days). Allowing for the incubation period, we would therefore expect to see an effect of SPDM on hospitalisations from around 1–2 weeks, and on ICU admissions from around 2 weeks after SPDM were introduced. Both hospital admission rates and ICU admission rates began to decline from 4 April 2020 (end of week 14) and have since continued to decline (Figure 7 with further details in Supplementary Figure S7). Similarly, positivity rates through laboratory reports began to decline from 6 April 2020 (start of week 15) (further details in Supplementary Figure S8).

#### Mortality

**Figure 8:**
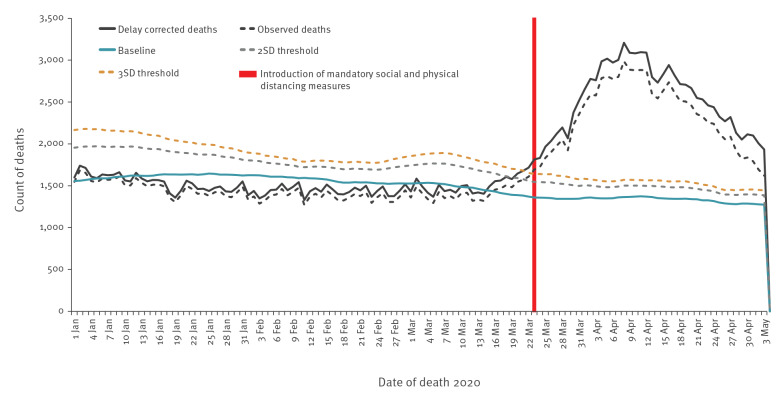
All cause mortality, all ages, United Kingdom, week 1 2020 to week 18 2020

Enhanced surveillance data on 25 deaths among the first FF100 in the UK indicated a median of 13 days between symptom onset and death (IQR: 7–19 days) [13]. Factoring in the incubation period we would therefore expect to see an effect of SPDM on mortality rates after 2–3 weeks. Deaths among both COVID-19 confirmed cases and excess all-cause mortality began to decline from 9 April 2020 (week 15), which is consistent with an impact of SPDM (Figure 8 and Supplementary Figure S9).

#### Summary of time to detectable impact

Time from the introduction of mandatory SPDM on 23 March 2020 to date of first detectable impact in each of the surveillance systems is summarised in the Table.

**Table:** Time from the introduction of mandatory social and physical distancing measures on 23 March 2020 to date of first detectable impact in each surveillance system, United Kingdom, week 1 2020 to week 18 2020

Surveillance system	Indicator	Frequency	Date of first detectable impact (2020)	Week of first detectable impact (2020)	Time to first detectable impact
Population survey	Self-reported fever or cough	Weekly	NA	13	< 1 week
Web search queries	Searches for COVID-19 symptoms	Daily	28 Mar	13	5 days
Syndromic	GP in hours COVID-19 indicator^a^	Daily	4 Apr	14	12 days
	GP out of hours ILI consultations	Daily	30 Mar	14	7 days
	GP out of hours difficulty breathing consultations	Daily	25 Mar	13	2 days
	GP out of hours ARI consultations	Daily	10 Mar	11	-12 days^b^
	A&E COVID-19 indicator^c^	Daily	6 Apr	15	14 days
	A&E ARI attendances	Daily	5 Apr	14	13 days
Primary care sentinel swabbing	GP sentinel swab SARS-CoV-2 positivity	Weekly	NA	13	< 1 week
Respiratory outbreaks	ARI outbreaks	Weekly	NA	16	4 weeks
Secondary care	COVID-19 hospital admissions	Daily	4 Apr	14	12 days
	COVID-19 critical care admissions	Daily	4 Apr	14	12 days
	Laboratory SARS-CoV-2 positivity rates	Daily	6 Apr	15	14 days
Mortality	Deaths among COVID-19 confirmed cases	Daily	9 Apr	15	17 days
	Excess all-cause mortality	Daily	9 Apr	15	17 days

A&E: accident and emergency department; ARI: acute respiratory infection; COVID-19: coronavirus disease; GP: general practice; ILI: influenza-like illness; NA: not available; SARS-CoV-2: severe acute respiratory syndrome coronavirus 2.

^a^ Indicator includes consultations using new codes for suspected, tested, exposed and confirmed COVID-19.

^b^ Detected 12 days prior to the start of mandatory social and physical distancing measures.

^c^ With a COVID-19 related primary diagnosis code.

## Discussion

Evidence from a multi-tiered surveillance system suggests that the mandatory SPDM in March 2020 have had a clear impact on COVID-19 activity during the first wave in England. This was first detectable as a reduction in self-reported relevant symptoms and presentations to community healthcare services, followed by a reduction in hospitalisations and critical care admissions and subsequently a reduction in deaths among COVID-19 confirmed cases and all-cause mortality. The timing of these reductions is generally in line with the expected intervals between infection and the respective outcome measures (Figure 1).

Most European countries have similar multi-tiered surveillance systems for COVID-19, which are often based on longstanding influenza surveillance mechanisms. The findings of this study will therefore be broadly applicable. In April 2020, the European Centre for Disease Prevention and Control (ECDC) provided guidelines on COVID-19 surveillance strategies and how these may be achieved. This highlights the need for comprehensive surveillance systems that can monitor intensity, geographic spread and severity of outbreaks [20], and the need for community-based surveillance through surveys, helplines, sentinel syndromic primary care surveillance, hospital-based severe acute respiratory infection (SARI) surveillance and mortality surveillance. Similar guidelines were subsequently issued by the World Health Organization [21]. Our study highlights how changes in disease activity can be detected through multi-tiered surveillance systems, which will help to inform future iterations of COVID-19 surveillance strategies guidance

Some of the changes in disease activity started earlier than was anticipated, so were unlikely to be a result of the mandatory SPDM. These changes could have been due to earlier government advice on isolating when displaying possible COVID-19 symptoms and reducing contacts with others, changes in behaviour independent of formal guidelines, or may reflect the distribution in timing between infection and different outcomes (i.e. a proportion of individuals will have shorter incubation periods and present earlier to healthcare services) [22]. The effect of the SPDM on acute respiratory infection outbreaks in nursing homes has been less marked. Investigations are ongoing to gain a greater understanding of the factors influencing transmission in nursing homes. The SPDM introduced may have a more limited impact in residential settings where infection could have been introduced by staff movements, or to a lesser extent following hospital discharge or nursing home transfer, and where it may be difficult to contain transmission through infection, prevention and control measures once infection is introduced [23]. Furthermore, there may be delays in reporting from nursing homes.

A large number of modelling studies have been undertaken to predict the impact of SPDM on COVID-19 activity and these have informed government policy around the world [24-27]. Conversely, few studies have evaluated the impact of these measures and the effectiveness of surveillance systems in detecting changes in disease activity. These studies have focussed on the impact on detected confirmed cases, primarily through testing in secondary care, and the impact on deaths among confirmed cases, again primarily in hospitals. A clear impact of social and physical distancing measures on these outcomes has been observed in China and several European countries [28-31]. In Hong Kong, the impact of SPDM on influenza detected through sentinel outpatient swab positivity and hospitalisations has also been assessed as a proxy for COVID-19. Here, SPDM were associated with declines in both these indicators [32].

The ability of surveillance systems to rapidly detect changes in COVID-19 activity is important. Each time countries begin to relax their SPDM, surveillance systems need to be carefully monitored to detect changes in activity. Our findings highlight the importance of symptom surveys in the population and syndromic surveillance as early indicators of COVID-19 activity. Nevertheless, existing indicators used in these systems are not specific to COVID-19. In the UK, the 2019/20 influenza season was relatively early which is likely to have increased the specificity of symptomatic and syndromic surveillance indicators. These measures are likely to be much harder to interpret when other seasonal respiratory viruses are circulating. There are also further limitations with symptoms surveys, such as recall bias, though given that the survey referred to in this analysis is conducted weekly this is unlikely to be a major issue. COVID-19 specific indicators of community transmission, including sentinel swabbing, will therefore be increasingly important. Moving to postal nasal self-swabbing has been successfully adopted in sentinel practices in England since February 2020 and overcomes many infection control concerns [33]. However, the push to offer wider access to SARS-CoV-2 testing based on patient demand from March 2020 risked undermining the consistency of the surveillance based on primary care consultations, the potential to test for a range of respiratory viruses, access to linked information on vaccination and the central source of specimens for further characterisation.

Some elements of the surveillance have been difficult to interpret because of changes in care pathways and coding. In particular, this has impacted on syndromic surveillance systems, which have now been enhanced to capture new COVID-19 clinical codes and activity. These new codes include NHS 111 calls and online assessments, GP attendances, emergency department attendances and ambulance calls. Attendances at GP surgeries have been reduced in favour of telephone consultations to maintain effective infection control. This impacted on the existing programme of primary care sentinel swabbing. To overcome this, self-sampling by post was initiated. Further expansion of this network continues to monitor the impact of any relaxation of restrictions.

There are still limited surveillance data available on asymptomatic infection. All the surveillance systems outlined in this paper would not routinely capture asymptomatic infection. Current evidence suggests that a large proportion of infections result in mild disease or asymptomatic infection. A cross-sectional swabbing of 948 London residents organised by PHE at the end of March identified 18 individuals positive for SARS-CoV-2 of which 4 (22%) had not reported any symptoms in the preceding 2 weeks (data not shown). These may represent people who are genuinely asymptomatic, those who are pre-symptomatic or those who remain PCR positive more than 2 weeks after infection. Asymptomatic infection will not be captured through community-based syndromic surveillance systems or sentinel swabbing of patients presenting to healthcare, and will rely on repeat population-based swabbing and seroprevalence estimates. Serum samples for serosurveillance have been collected since the early stages of the COVID-19 epidemic including residual samples from regional laboratories, samples from the RCGP RSC practices collected from patients presenting for other routine bloods and a prospective population-based collection in children and young adults. These are being tested and may provide a better understanding of the impact of SPDM on overall rates of infection.

### Conclusions

This analysis suggests a clear impact of the SPDM on COVID-19 activity in England, which was first detectable through community indicators within a week of the introduction of mandatory SPDM measures. We also highlighted the importance of community surveillance indicators for monitoring early changes in disease activity. Nevertheless, non-specific community indicators will become more difficult to interpret as circulation of other respiratory viruses increases. Syndromic surveillance indicators and population surveys should therefore be accompanied by community testing and case-based surveillance with robust mechanisms for capturing data on positivity rates, test indications and epidemiological characteristics of the population tested. Consistent, uninterrupted COVID-19 surveillance will be critical in monitoring the pandemic, informing the triggers for different phases and understanding the impact of relaxing SPDM and other interventions such as immunisation, as they come in to play.

## Supplementary Data

1383000 Supplement
