# Supplementary material for: The impact of social and physical distancing measures on COVID-19 activity in England: findings from a multi-tiered surveillance system
Source: Euro Surveill. 2021 Mar 18;26(11):2001062. doi: 10.2807/1560-7917.ES.2021.26.11.2001062 (PMC7976385; doi:10.2807/1560-7917.ES.2021.26.11.2001062)

This supplementary material is hosted by *Eurosurveillance* as supporting information alongside the article 'The impact of social and physical distancing measures on COVID-19 activity in England: findings from a multi-tiered surveillance system', on behalf of the authors, who remain responsible for the accuracy and appropriateness of the content. The same standards for ethics, copyright, attributions and permissions as for the article apply. Supplements are not edited by *Eurosurveillance* and the journal is not responsible for the maintenance of any links or email addresses provided therein.

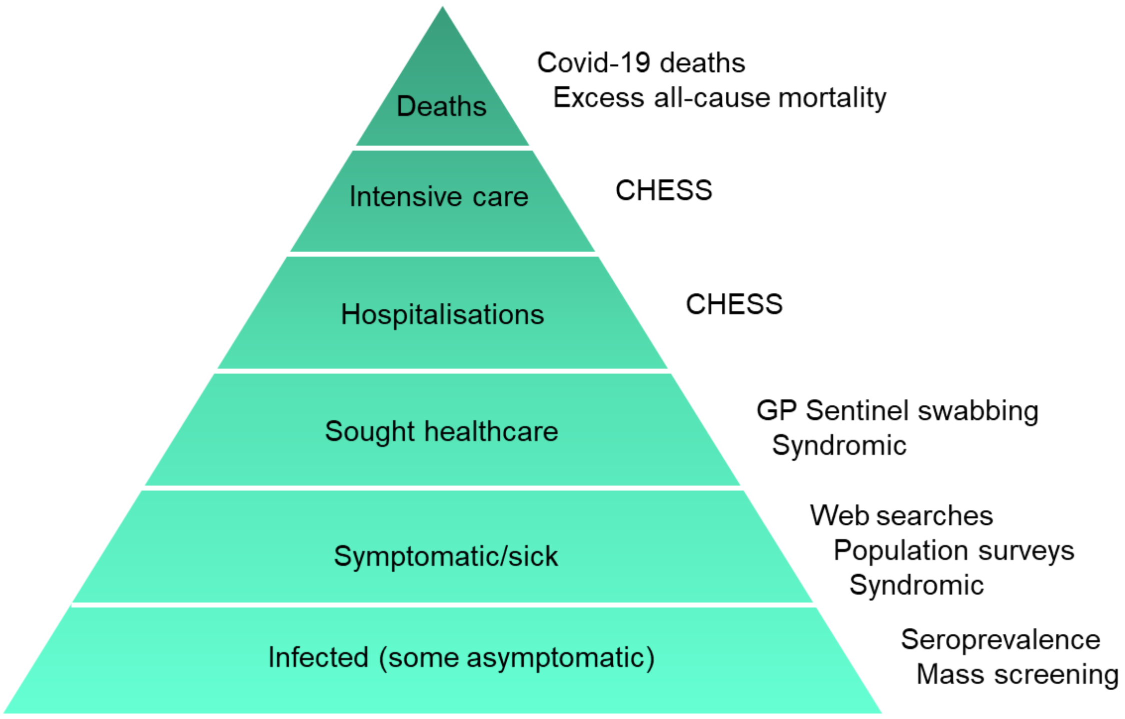

Supplementary Figure 1: COVID-19 Surveillance Pyramid

CHESS = COVID-19 Hospitalisations in England Surveillance System

Supplementary Figure 2: Rate of fever and cough among FluSurvey participants and their contact with different healthcare services, week 09 to 19. Red vertical line indicates introduction of mandatory SPDM

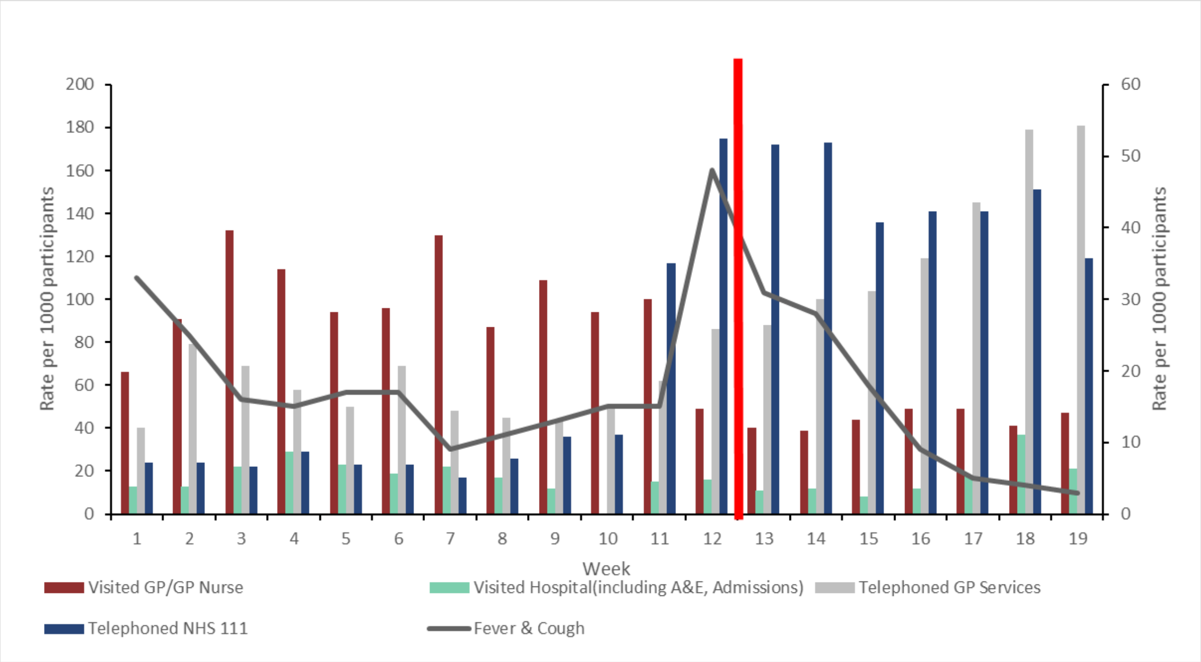

Supplementary Table 1: Time from symptom onset to first contact with health services (FluSurvey)

**Time from symptom onset to contact**	**Percentage accessing Health Service**					
	**Visited**			**Called**		
	**GP**	**Hospital Admission**	**ED**	**GP Reception**	**GP/Nurse**	**NHS111**
Same day	2.4	17.3	18	10	6.7	24.9
1 day	4.6	9.3	11.6	10.4	7	16.4
2 days	5.1	6.7	7.6	6.8	6.8	10.7
3 days	6.7	4	6.4	7.8	11.1	9.2
4 days	7.1	0	5.8	7.4	6.8	6.5
5-7 days	18.4	24	20.4	14.9	17.3	12.2
More than 7 days	52	33.3	29.1	40.8	41.4	16.8
don't know/can't remember	3.5	5.3	1.2	1.9	2.9	3.3
**Total Number of respondent**	**963**	**75**	**172**	**309**	**781**	**920**

Supplementary Figure 3: NHS 111 cough, daily calls, as a percentage of all calls (and 7-day moving average)

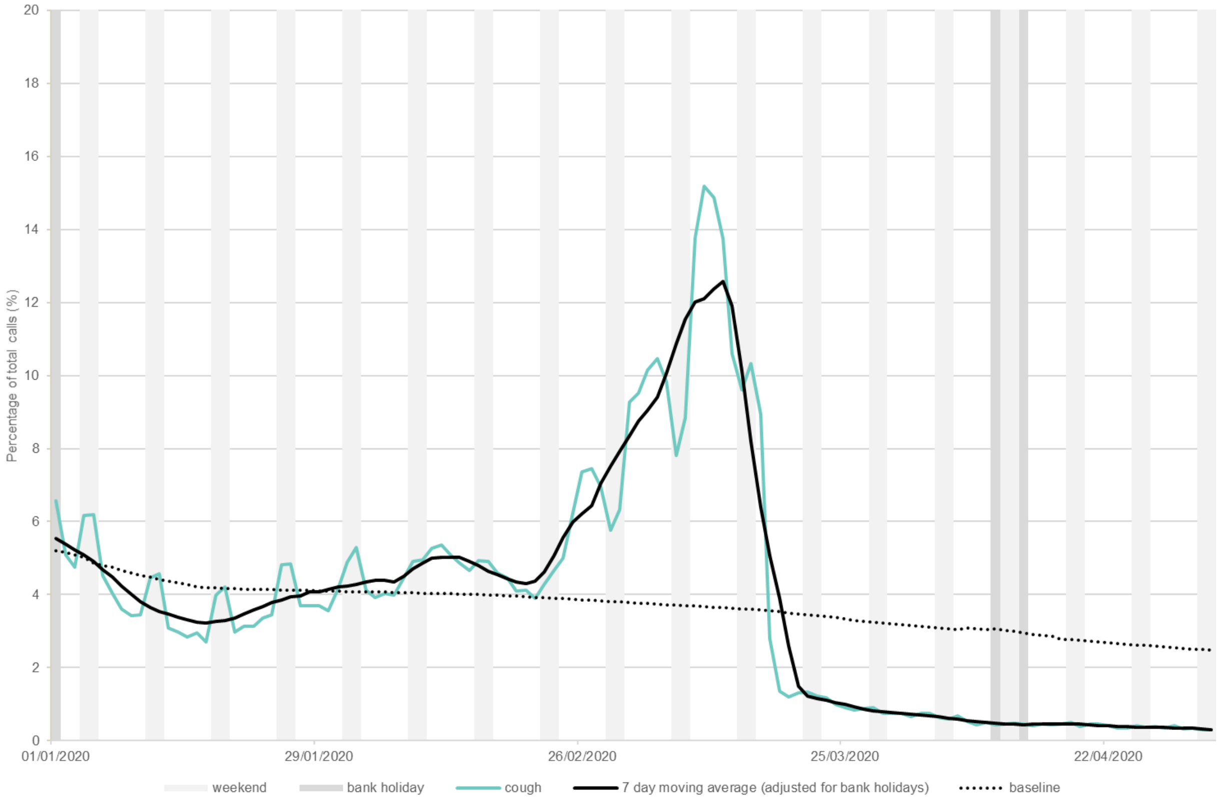

Supplementary Figure4: NHS 111 'potential COVID-19' (a) completed calls and (b) online assessments which have a 'potential COVID-19' final disposition (and 7-day moving average)

(a)

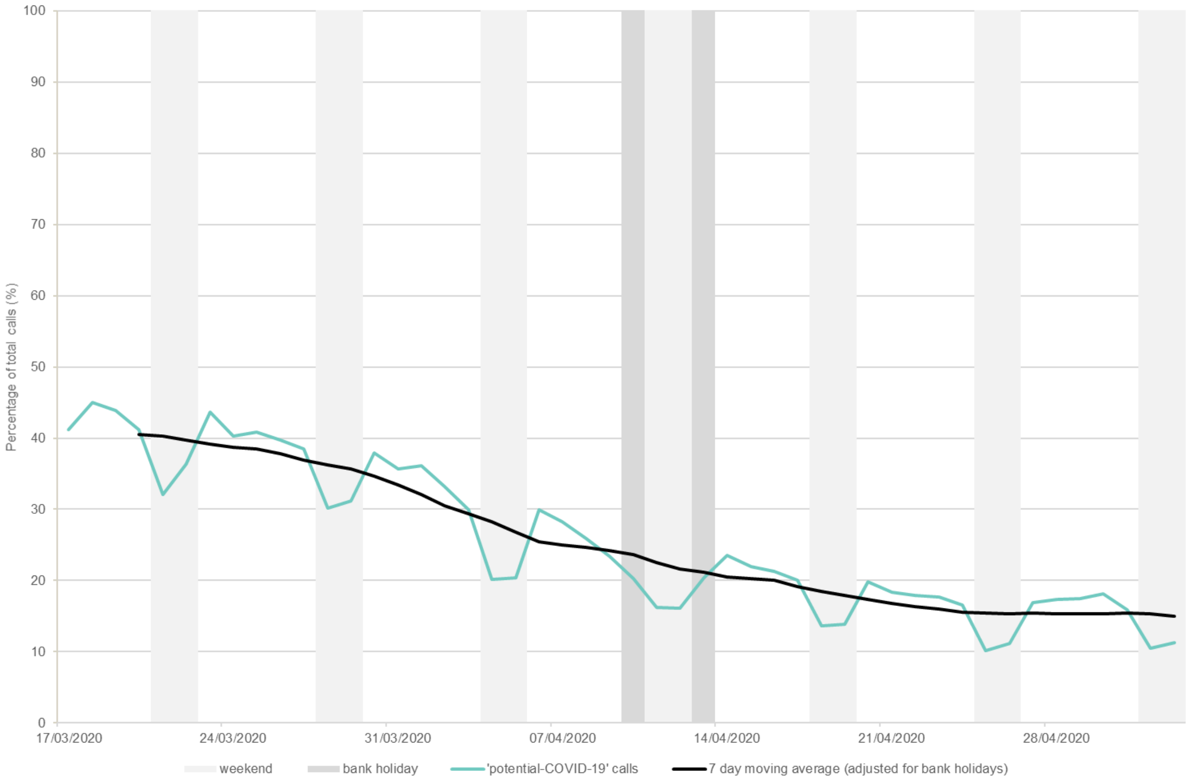

(b)

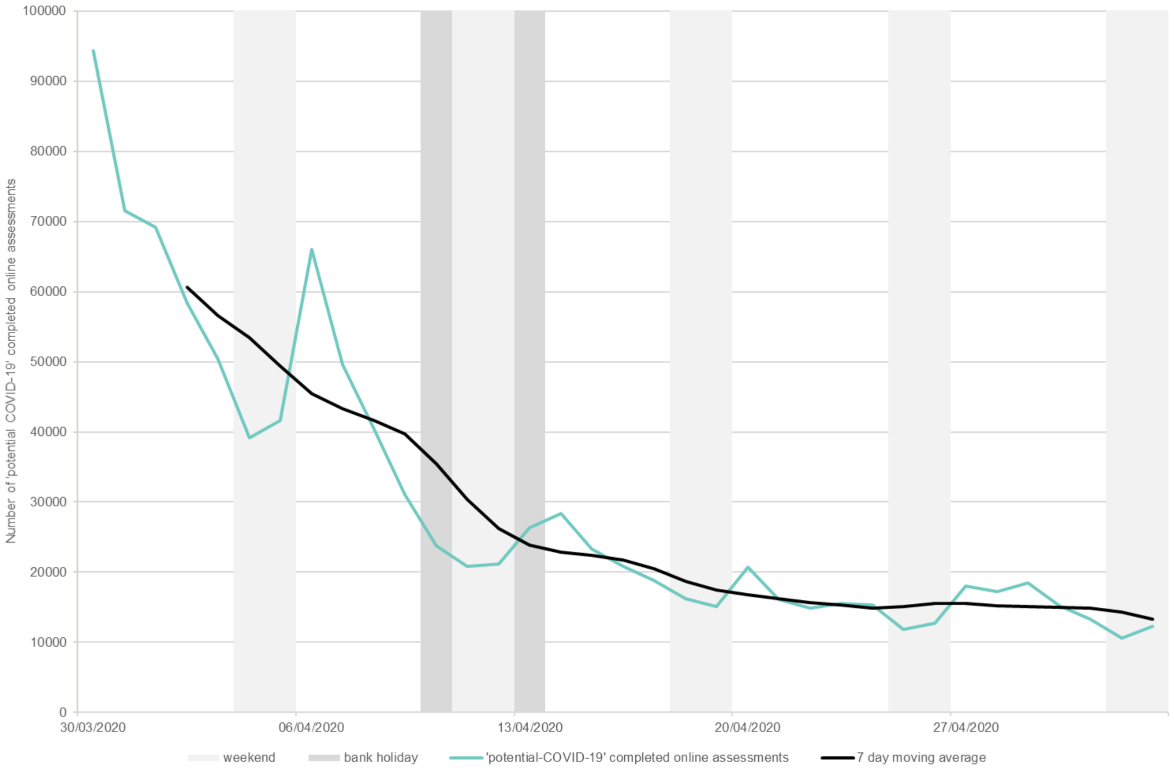

100000
90000
80000
70000
60000
50000
40000
30000
20000
10000
0
Number of 'potential COVID-19' completed online assessments
30/03/2020
06/04/2020
13/04/2020
20/04/2020
27/04/2020
weekend
bank holiday
'potential-COVID-19' completed online assessments
7 day moving average

Supplementary Figure 5: (a)ED COVID-19-like, daily attendances, as the number of attendances with a COVID-19 related primary diagnosis code (and 7-day moving average); (b) ED acute respiratory infection (ARI), daily attendances, as the number of attendances with an ARI related primary diagnosis code (and 7-day moving average)

(a)

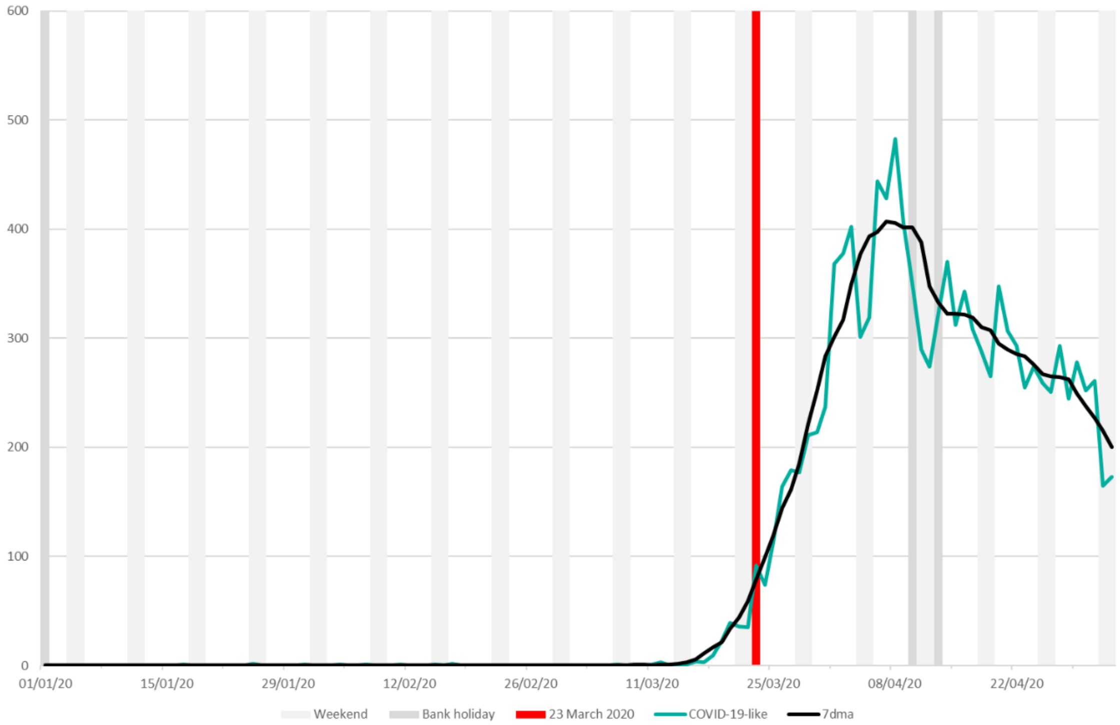

(b)

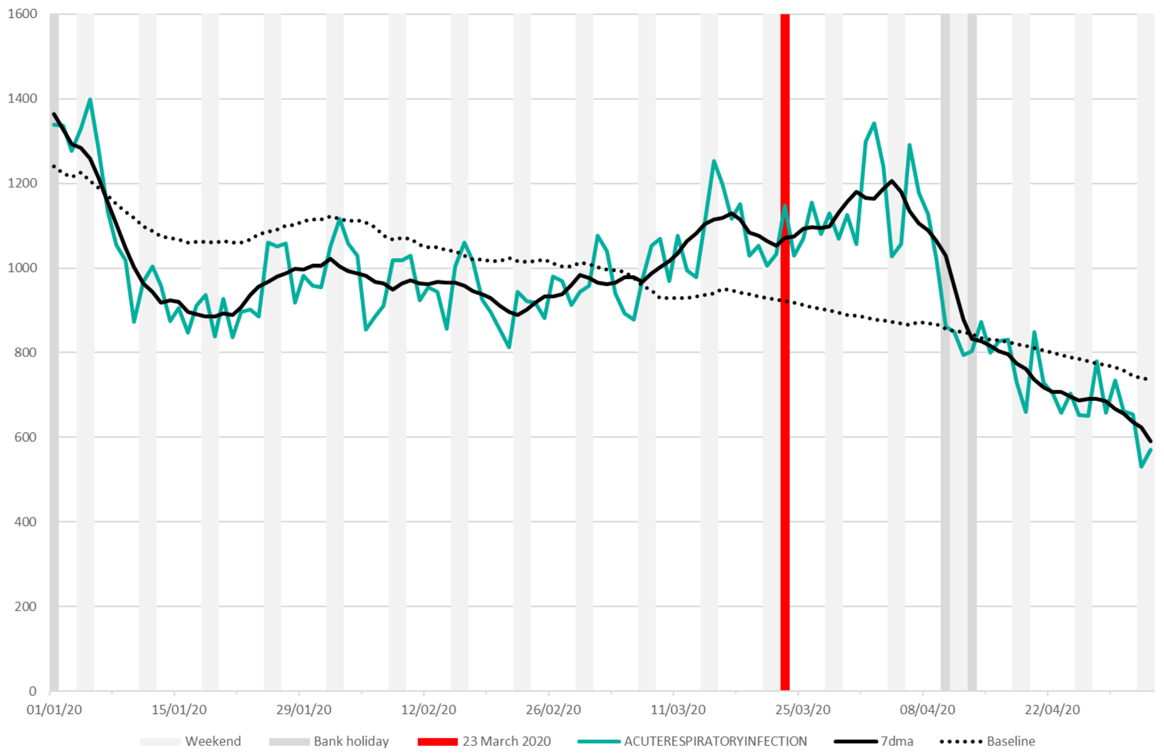
1600
1400
1200
1000
800
600
400
200
0
01/01/20
15/01/20
29/01/20
12/02/20
26/02/20
11/03/20
25/03/20
08/04/20
22/04/20
Weekend
Bank holiday
23 March 2020
ACUTERESPIRATORYINFECTION
7dma
Baseline

Supplementary Figure 6: Overall positivity (%) of GP sentinel swabs (a) by age group, (b) by sex (weekly)

(a)

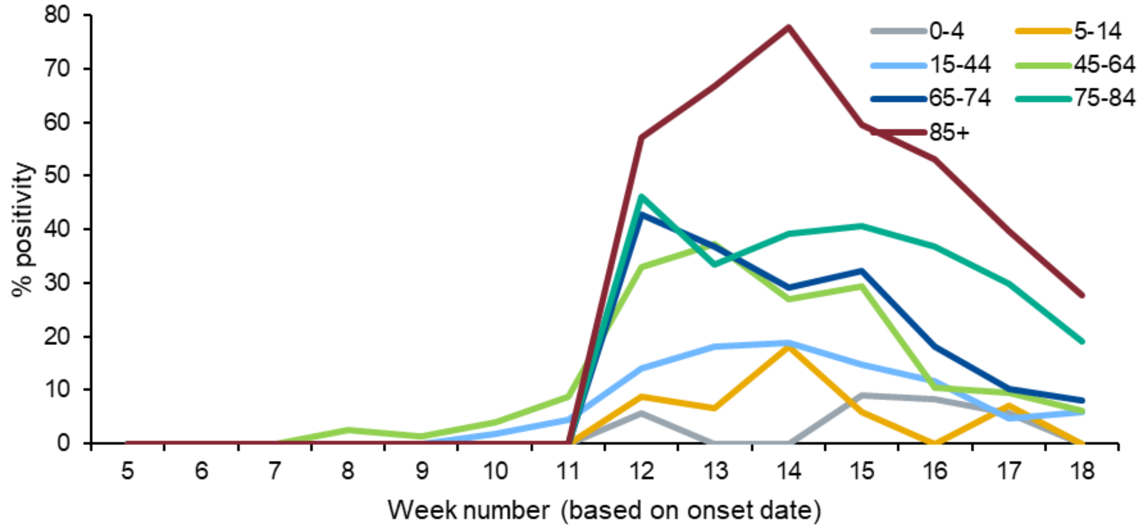

(b)

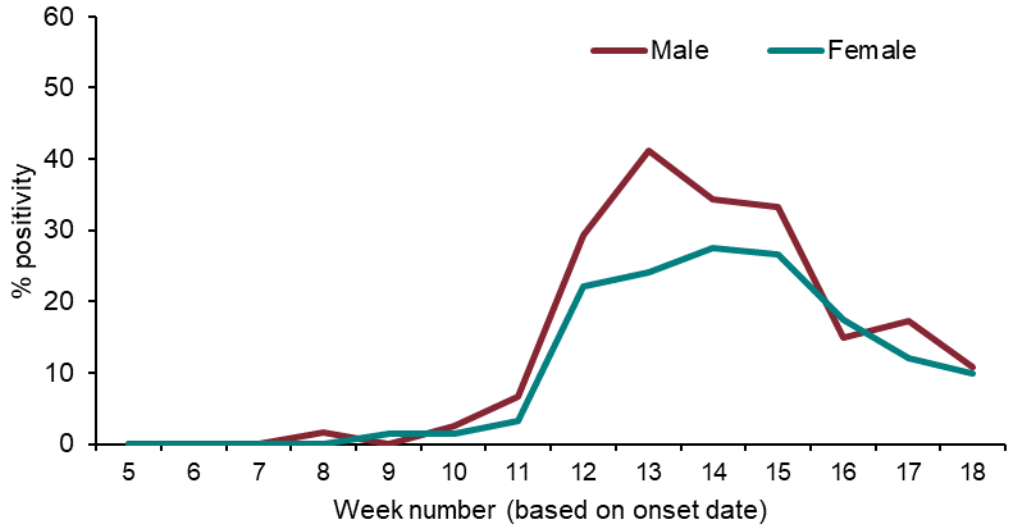

Supplementary Figure 7: (a) hospital admission rate and (b) critical care (ICU/HDU) admission rate by age group.

(a)

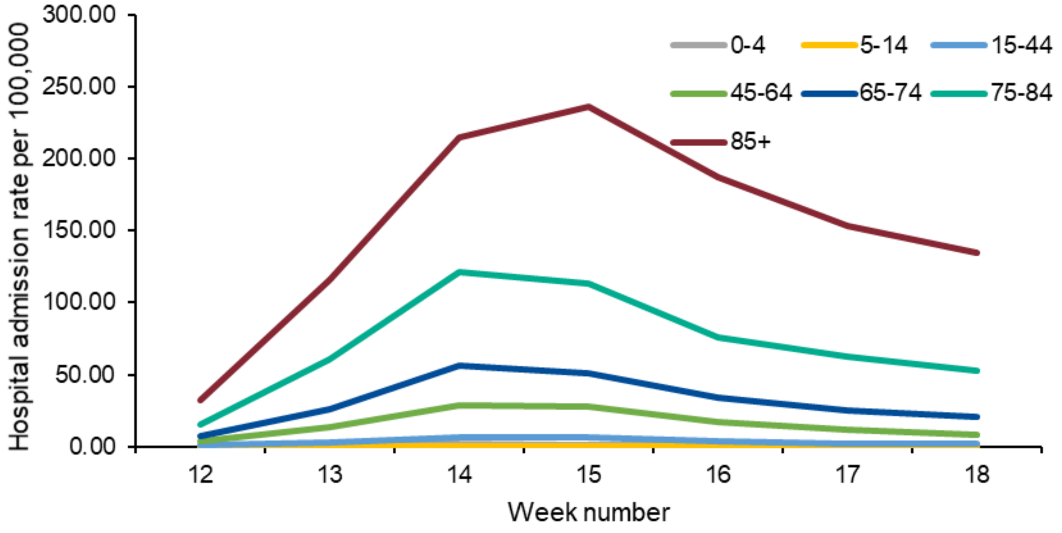

(b)

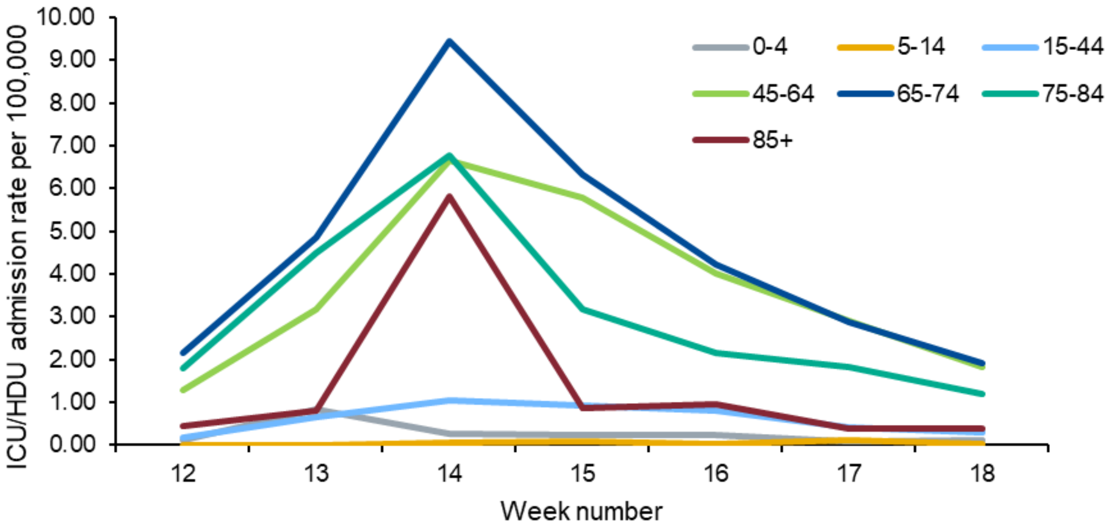

Supplementary Figure8: Overall positivity (%) (daily) and number of SARS-CoV-2 positive samples (SGSS)

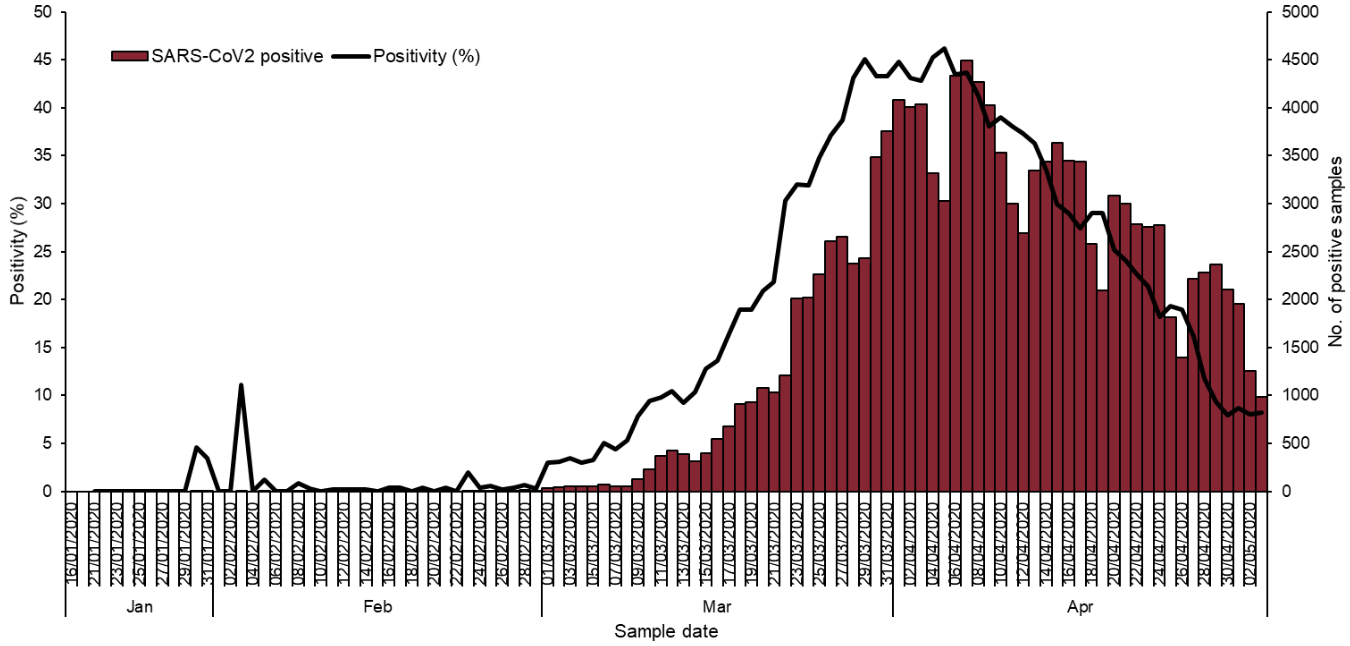

Supplementary Figure9: Cumulative number of deaths among COVID-19 confirmed cases by date of death and age group

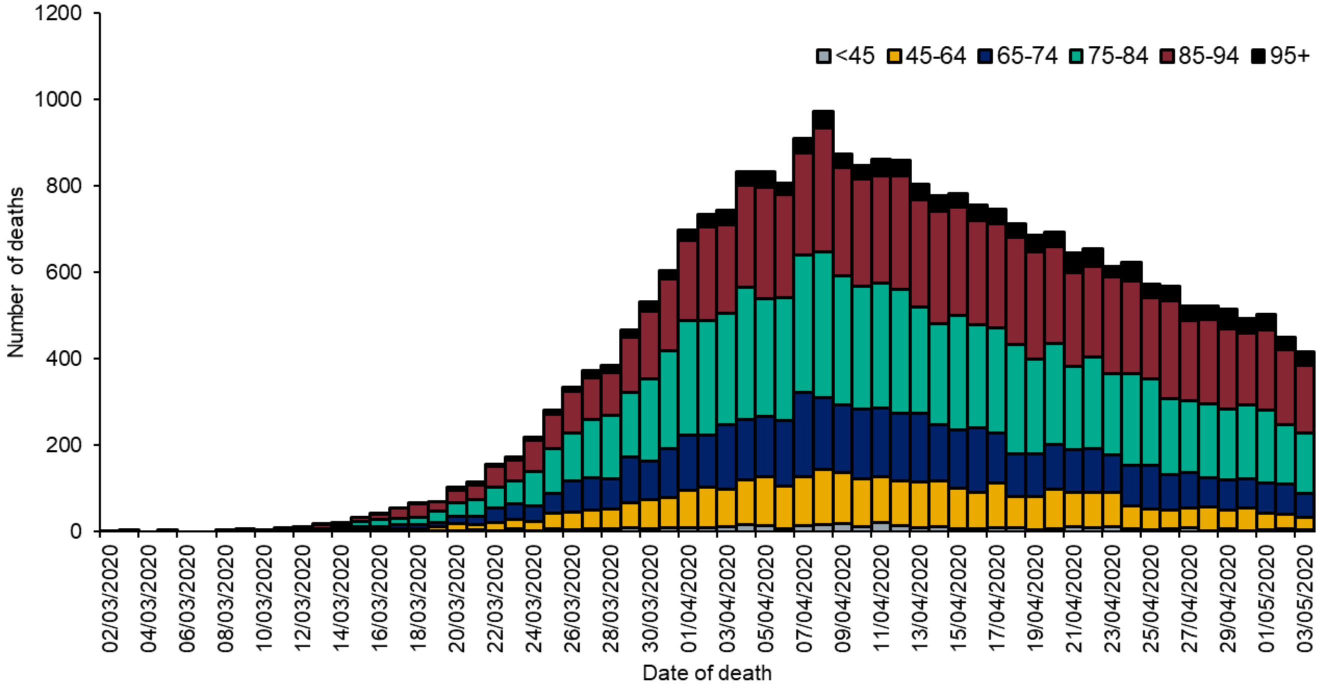

Supplement: Supplement [file 20-01062_BERNAL_Supplement.pdf]
